# Resting-State Fluctuations of EEG Sensorimotor Rhythm Reflect BOLD Activities in the Pericentral Areas: A Simultaneous EEG-fMRI Study

**DOI:** 10.3389/fnhum.2017.00356

**Published:** 2017-07-06

**Authors:** Shohei Tsuchimoto, Shuka Shibusawa, Nobuaki Mizuguchi, Kenji Kato, Hiroki Ebata, Meigen Liu, Takashi Hanakawa, Junichi Ushiba

**Affiliations:** ^1^School of Fundamental Science and Technology, Graduate School of Keio UniversityKanagawa, Japan; ^2^The Japan Society for the Promotion of ScienceTokyo, Japan; ^3^Department of Biosciences and Informatics, Faculty of Science and Technology, Keio UniversityKanagawa, Japan; ^4^Department of Rehabilitation Medicine, Keio University School of MedicineTokyo, Japan; ^5^Saiseikai Kanagawa-ken HospitalKanagawa, Japan; ^6^Integrative Brain Imaging Center, National Center of Neurology and PsychiatryTokyo, Japan; ^7^Japan Science and Technology Agency, Precursory Research for Embryonic Science and TechnologySaitama, Japan; ^8^Keio Institute of Pure and Applied SciencesKanagawa, Japan

**Keywords:** resting state, sensorimotor rhythm, alpha-band, beta-band, spontaneous fluctuation, pericentral area, simultaneous EEG-fMRI

## Abstract

Blockade of the scalp electroencephalographic (EEG) sensorimotor rhythm (SMR) is a well-known phenomenon following attempted or executed motor functions. Such a frequency-specific power attenuation of the SMR occurs in the alpha and beta frequency bands and is spatially registered at primary somatosensory and motor cortices. Here, we hypothesized that resting-state fluctuations of the SMR in the alpha and beta frequency bands also covary with resting-state sensorimotor cortical activity, without involving task-related neural dynamics. The present study employed functional magnetic resonance imaging (fMRI) to investigate the neural regions whose activities were correlated with the simultaneously recorded SMR power fluctuations. The SMR power fluctuations were convolved with a canonical hemodynamic response function and correlated with blood-oxygen-level dependent (BOLD) signals obtained from the entire brain. Our findings show that the alpha and beta power components of the SMR correlate with activities of the pericentral area. Furthermore, brain regions with correlations between BOLD signals and the alpha-band SMR fluctuations were located posterior to those with correlations between BOLD signals and the beta-band SMR. These results are consistent with those of event-related studies of SMR modulation induced by sensory input or motor output. Our findings may help to understand the role of the sensorimotor cortex activity in contributing to the amplitude modulation of SMR during the resting state. This knowledge may be applied to the diagnosis of pathological conditions in the pericentral areas or the refinement of brain–computer interfaces using SMR in the future.

## Introduction

Since Berger’s first electroencephalogram (EEG) recordings from the human scalp in the late 1920s, a number of studies have led to new insights into the function and mechanisms of intrinsic oscillations underlying brain activities (Niedermeyer and da Silva, 2005). One of the characteristic EEG rhythms is the sensorimotor rhythm (SMR), a spontaneous EEG (i.e., in the absence of somatosensory input or motor output) with an arch-shaped waveform in alpha (e.g., 7–11 Hz) and beta (e.g., 12–30 Hz) frequency bands (Kuhlman, 1978; Kozelka and Pedley, 1990; Arroyo et al., 1993; Kuhlman and Allison, 1977; Laufs et al., 2003; Hasan et al., 2016). The SMR signals are recorded from the C3 channel that is located closest to the hand area in sensorimotor area and its amplitude is known to be reduced by desynchronized neural activities in association with motor-related events, such as kinesthetic motor imagery or actual muscle contraction, suggesting a possible electrophysiological sign of sensorimotor excitability (Pfurtscheller et al., 2006). Actually, such SMR event-related desynchronizations (ERDs) are often localized over the pericentral gyri, and the degree of SMR-ERD is associated with cortico-spinal tract excitability (Takemi et al., 2013) as well as with intracortical disinhibition of the primary motor cortex.

Modulations of the SMR can be segregated into two physiologically different components. The alpha frequency band is located slightly posterior to the beta (Pfurtscheller and Lopes da Silva, 1999; Ritter et al., 2009) and shows prominent reaction to somatosensory events (Babiloni et al., 2008; van Ede et al., 2014). On the other hand, the amplitude of the beta frequency band EEG is attenuated, but the phase synchronization is maintained between the beta frequency band EEG and the spinal motoneuronal pool activities during tonic motor contraction tasks (Conway et al., 1995; Leocani et al., 1997; Bayraktaroglu et al., 2013). Furthermore, in the post-movement period, the amplitude of the beta-band consistently returns to and exceeds pre-movement levels via synchronization (Pfurtscheller et al., 1996a; Bauer et al., 2006; Jurkiewicz et al., 2006; Parkes et al., 2006). These studies suggest that the beta component of the SMR is more motor-related.

Does SMR also show some spontaneous fluctuations during the resting state, and, if so, is the resting-state SMR fluctuation correlated with blood-oxygen-level dependent (BOLD) signal changes in the sensorimotor areas? Spatial localization of alpha and beta components of the resting-state EEG-SMR has yet to be directly confirmed, but several lines of collateral evidence have supported such correlations. For instance, studies on sensory stimulation have shown that the prestimulus amplitude of the alpha-SMR has a significant impact on sensory stimulus detection (Linkenkaer-Hansen et al., 2004; Zhang and Ding, 2009). A few studies using single-pulse transcranial magnetic stimulation (TMS) over the primary motor cortex have shown an association between motor cortex activity or corticospinal tract excitability and the amplitude of the spontaneous beta-SMR during the resting state (Mäki and Ilmoniemi, 2010; Berger et al., 2014). Spontaneous fluctuations of the resting-state EEG may be much subtler than the motor task-related variations. However, similar to the fact that the level of the SMR synchronization reflects sensorimotor cortex activity during the course of a movement task (Pfurtscheller et al., 2000), the characteristics of the SMR may also be indicative of sensorimotor cortex activity during the resting state.

Here, based upon previous knowledge suggesting an association between spontaneous SMR fluctuation during the resting state and activity of the pericentral brain regions, we addressed the following hypotheses: (1) the spontaneous SMR power modulations were correlated with a surrogate marker of brain activity that covaries with resting-state sensorimotor cortical activity as measured by BOLD-fMRI in the pericentral area, and (2) the area correlated with alpha-band SMR was located posterior to the area correlated with beta-band SMR. To test such hypotheses, we employed EEG-fMRI simultaneous recording to identify the relationship between SMR modulations and whole-brain activity during the resting state.

## Materials and Methods

### Subjects

Nineteen healthy subjects (13 men and 6 women; aged 21–25 years) participated in this study. None had any sleep, medical, or psychiatric disorders. The purpose and experimental procedure were explained to the subjects, and all subjects gave informed written consent. The study was conducted in accordance with the Declaration of Helsinki and was approved by Keio University Faculty of Science and Technology Bioethics Committee and Saiseikai Kanagawa-ken Hospital Ethics Committee.

### Data Acquisition

The subjects were asked to lie still on a scanner bed in the dark for 10 min with their eyes opened and fixed on a small black cross. EEG signals were recorded with an MR-compatible amplifier (BrainAmp MR plus, Brain Products GmbH, Germany) and an EEG electrode cap (BrainCap MR, Brain Products GmbH, Germany) providing 63 EEG channels and 1 electrocardiogram (ECG) channel. Their electrodes were placed according to the modified International 10–10 system (Jurcak et al., 2007). The ground electrode was placed at the AFz, the reference electrode at the FCz, and the impedance of all electrodes was kept lower than 5 kΩ throughout the experiment. The ECG electrode was placed on the back of subjects to obtain the electrocardiographic data and to subsequently correct ballistocardiographic artifacts. Raw record was sampled at 5 kHz with a bandpass filter between 0.1 and 2500 Hz using the Brain Vision Recorder, (Brain Products GmbH, Germany). The amplifier system was set beside the subject’s head within the scanner during fMRI scanning.

Functional images were acquired on a 1.5-T MR scanner (Signa Excite, GE Medical Systems, United Kingdom) and whole-brain, T2-weighted BOLD data were acquired using an echo planar image sequence (repetition time TR = 3000 ms; echo time TE = 40 ms; flip angle = 70 degrees; voxel size = 3.75 mm × 3.75 mm × 4.0 mm; 30 axial slices with no gap, 200 scans). To achieve phase synchronization clocks for digital sampling between the MRI data and the EEG system, the EEG system clock was synchronized with a SyncBox device (Brain Products GmbH, Germany) and the MRI scanner’s 10 MHz master synthesizer. The scanner also delivered each TR trigger signal that marked the onset time of every fMRI volume acquisition. These markers were used for fMRI scanning artifact correction of the EEG data.

### Data Analyses

**FIGURE 1:**
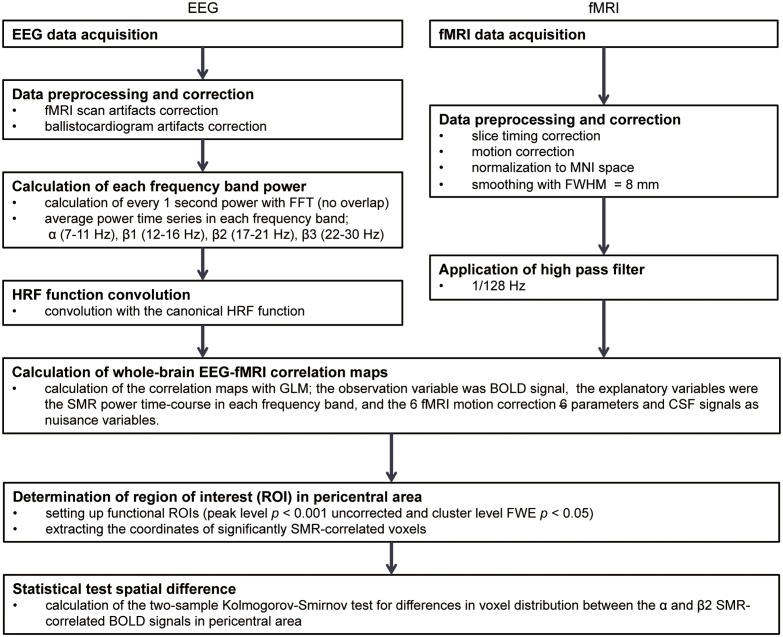
The analysis flow in EEG-fMRI simultaneous recording study. FFT, Fast Fourier transformation; HRF, hemodynamic responses; MNI, Montreal Neurological Institute; FWHM, full width at half maximum; GLM, General Linear Model; BOLD, blood-oxygen-level dependent; SMR, sensorimotor rhythm; CSF, cerebrospinal fluid; FWE, family-wise error.

An outline of the analyses is shown in **Figure 1**. The several-seconds scale of modulation in the SMR resting-state amplitude was first extracted from EEGs of healthy volunteers, and the SMR power time-series was convolved with the canonical hemodynamic response function (HRF) to model the BOLD signals co-varying with the SMR modulation. A voxel-wise correlation analysis was then conducted in the whole-brain to identify the cortical and subcortical activities with which the modulation of SMR amplitude was associated.

Electroencephalogram data were processed by Brain Vision Analyzer 2.0 (Brain Products GmbH, Germany) and the MR gradient-artifacts and ballistocardiogram in the EEG signals were corrected using the average template subtraction method (Allen et al., 2000). First, to create the gradient-artifact template, EEG data were averaged over three volumes (9 s) with the reference to each TR trigger signal. The template was computed for each TR interval using the sliding window. The gradient-artifact template was subsequently subtracted from the original EEG data. Second, the ballistocardiographic artifacts were removed in a similar way. The template in this case was constructed by averaging of the EEG data with the reference to the peaks of the R-waves in the ECG signal. After the gradient-artifacts and ballistocardiographic corrections, the EEG data was bandpass filtered (3–70 Hz with 48 dB/octave phase shift-free Butterworth) with a notch (50 Hz for avoiding power line contamination), and the sampling rate of the data was decreased to 250 Hz.

It is known that the EEG signal obtained around the pericentral area contains cognitive components, such as activity related to the “mirror neuron systems,” in addition to the sensorimotor idling activity component (Yin et al., 2016). Because we focused on the sensorimotor processing component, we obtained SMR signals from the C3 channel that is located closest to the pericentral area. Furthermore, the re-reference to the common average reference should remove global-signal to emphasize the relevant localized activities around the pericentral area (McFarland et al., 1997). The power time courses of EEG data in the C3 channel were calculated by a Fast Fourier Transform (FFT) with a 1-s moving time window and no overlap. We first selected four different frequency bands [7–11 Hz (alpha), 12–16 Hz (beta1), 17–21 Hz (beta2), 22–30 Hz (beta3)] as candidates for the sensorimotor-related features (Arroyo et al., 1993; Rangaswamy et al., 2002, 2004; Ritter et al., 2009), and the power time courses of SMR to represent the SMR amplitude fluctuations were obtained by calculating the band power in each time window by averaging the magnitudes of the Fourier coefficients in the related frequency bins. To confirm that these frequency components were localized over the sensorimotor cortex, simple linear regression coefficients at a zero time lag were calculated between the C3 and the other channels. The coefficients were assigned to each channel position where the correlation with C3 was calculated, and a topographic map was obtained using the nearest neighbor interpolation with the MATLAB 4 griddata method. After calculating the regression coefficients for each individual subject, a one-sample *t-*test was performed for group analysis.

The fMRI data were pre-processed with Statistical Parametric Mapping (SPM12, Wellcome Department of Imaging Neuroscience^1^) on MATLAB (MathWorks, Natick, MA, United States). First, the slice-timing correction was applied with the reference slice based on the middle of each TR, and each volume was spatially realigned with the first volume. After the timing and spatial corrections, each volume was normalized according to the Montreal Neurological Institute (MNI) template, and was resampled to 2-mm cubic voxels. The normalized volumes were then smoothed with an isotropic Gaussian kernel [Full width at half maximum (FWHM) = 8 mm], and were 1/128 Hz high-pass filtered.

For the correlation analysis of BOLD signals with SMR power modulation in each frequency band, we followed a previous approach in the simultaneous EEG-fMRI study (Huster et al., 2012). First of all, the SMR power time series for bandwidth was convolved with HRF provided by SPM12 to take into account the hemodynamic delay of the BOLD signal. After the convolution, to conform the temporal resolution of the fMRI data, the SMR power time courses were down-sampled with the data point sampled at the reference of the fMRI slice timing correction; namely, one data point was sampled in the middle of every TR. To identify the brain regions with correlations between BOLD signals and SMR fluctuations, each alpha and beta frequency band of SMR power was used as a predictor for the BOLD signal in the General Linear Model (GLM). Because the alpha- and beta-band SMR power modulations were significantly correlated, a GLM model was created for each frequency of SMR power to avoid multicollinearity. The six realignment parameters (three translation and three rotations) were used as nuisance variables to remove head motion artifacts, and the cerebrospinal fluid (CSF) signal was also used as a nuisance variable to exclude non-neural noise originating from cardiac effects and respiration. The CSF time series were obtained by averaging the signal over all voxels within the Lateral Ventricles Mask, which were anatomically defined by the SPM12 atlas.

In the first-level analysis, we generated regression coefficient contrast images of alpha- and beta-band SMR modulations for each subject. Then, for the second-level, group data analysis, the contrast images were fed into a one-sample *t*-test. For all data, the fMRI results were subjected to peak- and cluster-extent-based thresholding, the most popular thresholding method for dealing with the issue of multiple comparisons over many voxels (Woo et al., 2014). The initial voxel threshold was set to *p* < 0.001 uncorrected for multiple comparisons (this level was considered reasonably conservative against a false positive finding). Thus, clusters were considered significant if they survived an extent threshold of *p* = 0.05 family-wise error (FWE) corrected for multiple comparisons for statistical inference. In each region of sensorimotor cortex correlated with alpha- or beta-power fluctuations of the SMR, we performed statistical evaluation tests to validate that these correlated regions were spatially different using a two-sample Kolmogorov–Smirnov test.

## Results

### EEG Results

**FIGURE 2:**
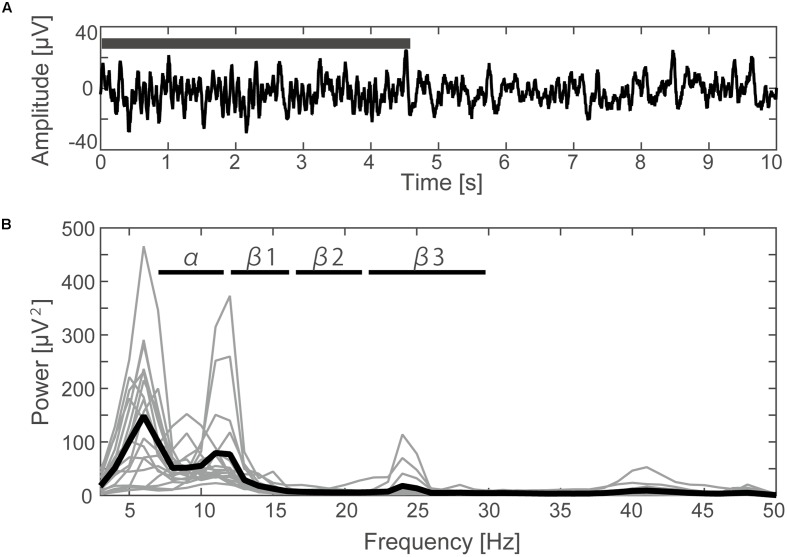
Time course and frequency results of the EEG. **(A)** Filtered EEG data (3–70 Hz with 48 dB/octave phase shift-free Butterworth bandpass filter and 50 Hz notch filter) elicited from the C3 channel of one subject. The black bar indicates the time period during which the typical SMR occurred. **(B)** The power spectrum density calculated by FFT for each subject (thin lines) and the averaged data from all subjects (thick line).

**FIGURE 3:**
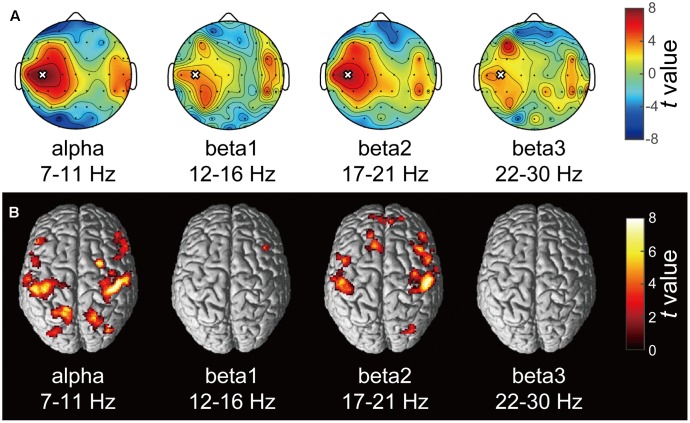
Correlation maps of the SMR modulation wave elicited from C3 in each frequency band. **(A)** Each topography map shows the spatial distribution of the correlation coefficient between the SMR modulation elicited from C3 and that at other electrodes. The white crosses represent the C3 channel and the black dots represent other EEG channels. The color bar shows the strength of correlation. **(B)** The statistical *t*-maps of fMRI negative correlations of each component of SMR modulation with the BOLD signals (*p* < 0.001 uncorrected, cluster-level FWE *p* < 0.05).

A typical SMR time series of a single subject is shown in **Figure 2A**, and the power spectrums of the SMRs in all subjects are depicted in **Figure 2B**. Although EEG signal power varied among the subjects, the averaged power spectrum density showed that the overall frequency structures were shared across the subjects, with the peak-frequencies at 10–12 Hz and 22–26 Hz. The averaged powers in the alpha and beta1 frequency band were 6.8 × 10^-5^ V^2^ and 2.9 × 10^-5^ V^2^, respectively. The powers of beta2 and beta3 were 6.0 × 10^-6^ V^2^ and 7.5 × 10^-6^ V^2^, respectively, and were relatively small in comparison with the former two frequency bands. For each subject, the power-modulation-depth ratios were calculated as coefficients of variation; namely, the ratio of the standard deviation (SD) to the mean of time-fluctuation. The inter-subject average and SD of the power-modulation-depth ratios were 85 ± 16% in alpha; 112 ± 20% in beta1; 88 ± 39% in beta2 and 68 ± 19% in beta3 during the resting state. **Figure 3A** shows the spatial distribution of the selected frequency modulation components from C3. All of the distribution maps showed positive correlations locally around C3 and its contralateral (C4) electrode, with notable localization in the alpha and beta2 components.

### fMRI Results

**Figure 3B** depicts the statistically significant *t*-maps of fMRI correlates (BOLD signals) of SMR modulation, which were assessed separately for the alpha and each beta component. The alpha-band SMR modulation was negatively correlated with the activities of the bilateral pericentral cortices (*p* < 0.001 peak level uncorrected; *p* < 0.05 voxel level FWE corrected). The *t*-values and MNI coordinates are shown in **Table 1**. Similar to the correlation of BOLD signals with the alpha-band SMR modulation, the beta2-band SMR modulation was negatively correlated with the activities of the bilateral pericentral cortices (**Table 2**). The beta3 SMR modulation was not correlated with any region. The beta1 SMR modulation was negatively correlated with the BOLD signal in the right middle frontal gyrus; this correlation was also seen in the analysis of the alpha- and beta2-band SMR modulations.

**Table 1:** Brain regions whose activity correlated with the power of the alpha-band SMR modulation.

Model	Correlation type	Brain region	Side	MNI coordinates			*t*-score	*p*-value	Cluster size
							
				*x*	*y*	*z*			
Alpha	Negative	Triangular part of the inferior frontal gyrus	R	50	38	-6	5.95	6.30 × 10^-5^	316
		Postcentral gyrus	L	-52	-32	52	5.88	7.23 × 10^-6^	2002
			R	46	-28	62	5.85	7.61 × 10^-6^	1250
		Middle frontal gyrus	L	-44	28	28	5.57	1.36 × 10^-5^	430
		Superior parietal lobule	R	22	-68	50	5.53	1.50 × 10^-5^	791
			L	-16	-58	64	5.25	2.72 × 10^-5^	642
		Superior frontal gyrus	R	26	2	62	5.25	2.73 × 10^-5^	241
		Opercular part of the inferior frontal gyrus	R	56	22	16	5.03	4.34 × 10^-5^	838
		Middle occipital gyrus	L	-34	-84	30	4.54	1.27 × 10^-4^	202


*Brain regions are named based on the SPM12 atlas (Statistical Parametric Mapping, Wellcome Department of Imaging Neuroscience, http://www.fil.ion.ucl.ac.uk/spm) *p*-value, cluster-level family-wise error rate of 0.05. MNI, Montreal Neurological Institute.*

**Table 2:** Brain regions whose activity correlated with the power of the SMR beta1- and beta2-bands.

Model	Correlation type	Brain region	Side	MNI coordinates			*t*-score	*p*-value	Cluster size
							
				*x*	*y*	*z*			
Beta1	Negative	Middle frontal gyrus	R	38	20	30	6.38	2.61 × 10^-5^	314
Beta2	Positive	Thalamus	R	2	-12	4	4.42	1.65 × 10^-4^	270
			L	-6	-8	6	3.71	8.06 × 10^-4^	270
	Negative	Superior frontal gyrus	L	-10	24	58	6.51	2.00 × 10^-6^	326
			R	22	54	30	4.72	8.47 × 10^-5^	202
		Postcentral gyrus	L	-44	-28	50	6.33	2.86 × 10^-6^	1262
			R	52	-18	52	5.94	6.41 × 10^-6^	1024
		Opercular part of the inferior frontal gyrus	R	42	14	24	6.09	4.64 × 10^-6^	1758
		Posterior orbital gyrus	R	32	24	-18	5.88	7.22 × 10^-6^	313
		Superior temporal gyrus	L	-60	-20	2	5.40	1.98 × 10^-5^	293
		Lingual gyrus	L	-20	-68	-10	5.01	4.50 × 10^-5^	219
			R	20	-66	-4	4.68	9.34 × 10^-5^	376
		Middle occipital gyrus	R	32	-82	32	4.72	8.52 × 10^-5^	224
		Superior frontal gyrus medial part	R	6	50	18	4.58	1.17 × 10^-4^	340
		Middle temporal gyrus	R	60	-30	0	4.40	1.73 × 10^-4^	228


*Brain regions are named based on the SPM12 atlas. *p*-value, cluster-level family-wise error rate of 0.05. MNI, Montreal Neurological Institute.*

**FIGURE 4:**
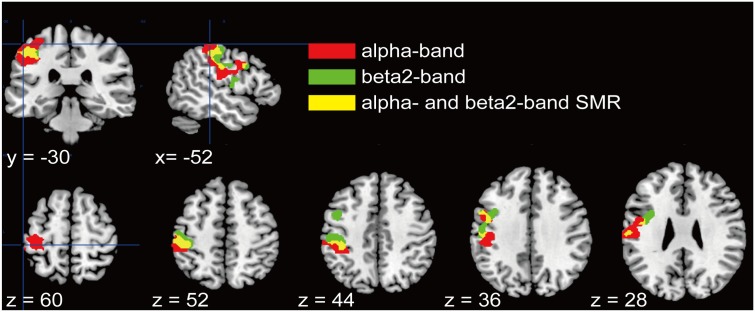
Spatial distributions of the correlations between BOLD signal and each SMR modulation in the pericentral area. Red areas correlated with alpha-band SMR modulation, green areas correlated with beta2-SMR modulation, and yellow areas are overlapped regions correlated with modulations of both alpha- and beta2-bands. These regions were adjusted from statistical maps to those based on the atlas of the pericentral area in SPM12.

**FIGURE 5:**
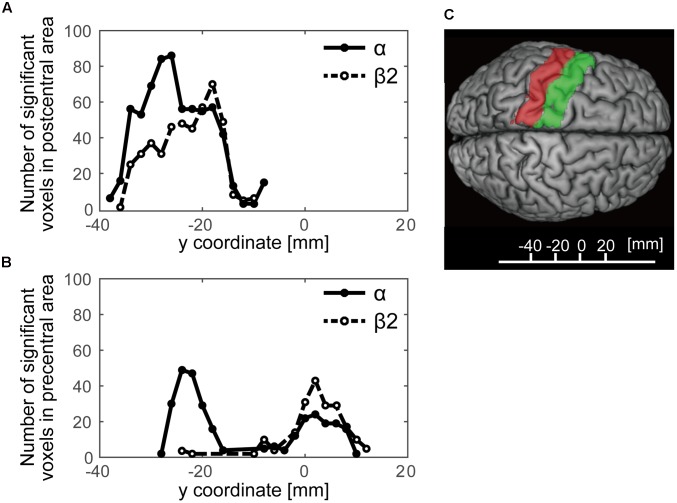
The distributions of statistically significant voxels along the anterior–posterior axis. The black lines show the number of significant voxels correlated with alpha-band SMR modulation and the dashed lines show those correlated with beta2-band SMR modulation. **(A)** The distribution of significant voxels in the postcentral area; **(B)** their distribution in the precentral area; **(C)** the *y*-axis Montreal Neurological Institute coordinates superimposed on the SPM12 atlas showing the postcentral (red) and precentral (green) areas.

We further examined a spatial topography of fMRI signal correlated with both the alpha- and beta2-band SMR modulations in the left pericentral area, where the C3 electrode was located. **Figure 4** shows the pericentral activity correlated with alpha-band SMR, beta2-SMR, and overlap of both. The peak coordinate of the alpha-band SMR-correlated activity was MNI (*x*, *y*, *z*) = (-52, -32, 52) and that of the beta2-band SMR was MNI (*x*, *y*, *z*) = (-44, -28, 50). Furthermore, **Figure 5** displays the distribution of these correlated areas along the anterior–posterior (*y*) axis. The spatial distributions of alpha-SMR and beta2-SMR correlated activities were different in both the postcentral (**Figure 5A**) and precentral (**Figure 5B**) areas (*p* < 0.001, two-sample Kolmogorov–Smirnov test). More precisely, the alpha-band SMR-correlated BOLD signals were distributed posterior to those correlated with the beta2-SMR.

## Discussion

In the present study, we employed simultaneous EEG-fMRI recordings in healthy human subjects to investigate the whole-brain hemodynamic responses associated with spontaneous SMR fluctuations. We found significant correlations between BOLD signals of activities in the sensorimotor regions and modulation of EEG-SMRs in the resting state. In addition, the regions with negative correlations between BOLD signals and the alpha-band SMR were distributed posterior to those correlated with the beta-band SMR.

### SMR Frequency Components in Alpha- and Beta-Bands

Two of the four frequency components, alpha- and beta2-band, showed distributions of significant correlations locally around C3 and its periphery. According to Ritter et al. (2009) study using a blind source separation algorithm, the SMR power modulations of the alpha (∼10 Hz) and beta (∼20 Hz) frequency bands were associated with the motor task. Thus, the amplitude modulation of the alpha- and beta2-bands of the SMR may reflect fluctuations in activity in the sensorimotor regions. Moreover, both the alpha- and beta2-band SMR components were correlated with BOLD signals around the bilateral pericentral areas, that periphery area of around C3 as well as the contralateral C4 area. The homolog C4 of bilateral pericentral areas, which is consistent with the fact that the two cerebral hemispheres have anatomical connections via the corpus callosum and each hemisphere communicates with the contralateral hemisphere by inhibitory and excitatory inputs (Bloom and Hynd, 2005; Palmer et al., 2012).

### Correlation between Intrinsic SMR Fluctuations and BOLD Signals

Both the alpha- and beta2-band SMR components were correlated with BOLD signals around the bilateral pericentral areas. Previous fMRI studies also found that in the resting state, BOLD signals in the bilateral sensorimotor cortices were correlated with one another, a phenomenon referred to as the *sensorimotor network* (Biswal et al., 1995; Beckmann et al., 2005). Furthermore, the alpha or beta synchronizations are interpreted as correlates of “idling” motor cortex neurons (Pfurtscheller et al., 1996b; Ritter et al., 2009). Therefore, the intrinsic alpha- and beta-band SMR modulations might reflect the sensorimotor network in the resting state. In addition, another simultaneous EEG-fMRI study showed that alpha- and beta-band SMR modulations were negatively correlated with the sensorimotor network (Yin et al., 2016). Our results are compatible with these previous findings, suggesting that it is valid to draw correlations between the intrinsic SMR modulations and activity in the sensorimotor cortex.

### Functional Properties of Alpha- and Beta-Band SMR

Although the topographic map of the EEG alpha-band SMR correlation was similar to that of the beta2 correlation, it may reflect somatosensory-dominant activities of the cortex. Our fMRI results may indicate functional differences between the alpha- and beta-band components of the SMR. Although both SMR components were correlated with BOLD signals around the pericentral area, the area correlated with the alpha-band SMR was in the parietal region and that correlated with the beta-band SMR was the frontal region. Recent findings suggest that the functional role of alpha oscillations is closely related to the activity of intrinsic cortical networks, indicating that the alpha-band signal occurs in different cortical layers (Klimesch et al., 2007; Palva and Palva, 2007; Ben-Simon et al., 2008; Bazanova and Vernon, 2014; Sigala et al., 2014). Activities in the beta-band, however, are known to be connected with motor functions (Brovelli et al., 2004; Jurkiewicz et al., 2006; Tzagarakis et al., 2015). In addition, in a combined TMS-electromyography (EMG) and concurrent EEG recording study, the Farzan group reported that EEG components correlated with the TMS-induced silence period in EMG, and that the cortical inhibitory processes in humans were related to the power of beta oscillations more locally than to that of the alpha oscillations (Farzan et al., 2013).

### Spatial Distribution of Alpha- and Beta-Bands SMR

Previous studies reported the SMR to be most prominent in central scalp regions in the area of the sensorimotor cortex (Pfurtscheller and Neuper, 1997; Birbaumer et al., 2000; Blankertz et al., 2010), while our results depict the spatial differences of correlation areas in intrinsic alpha- and beta-bands of the SMR. In studies of SMR power modulation associated with motor execution or motor imaginary tasks, the attenuated alpha- and beta-band SMR amplitudes were localized around the contralateral sensorimotor area (Crone et al., 1998; Sauseng et al., 2013). Furthermore, the center of ERD of the alpha-band SMR was located more posterior than that of the beta-band SMR in EEG/MEG studies (Pfurtscheller and Lopes da Silva, 1999; Jurkiewicz et al., 2006). The task-related reactive sources of the alpha-band SMR were localized in the postcentral area and those of the beta-band SMR were in the precentral area during sensory input (Cheyne et al., 2003) or motor output (Salmelin et al., 1995). Although it is not obvious that the neural processing of intrinsic SMR modulation during the resting state is equivalent to that of event-related task modulation, our results indicate that the spontaneous modulations of the SMR may be based upon a similar mechanism with that of the SMR modulated by sensory inputs or movements which were already shown by event-related studies to represent pericentral activities; thus the SMR modulation in the resting state is also likely to reflect pericentral activities. Therefore, insight into the relationship between SMR modulation and the pericentral area, which has been examined in event-related studies, has now been extended to the activities of the intrinsic SMR modulation during the resting state. In the present study, neural activity correlated with SMR modulation at rest showed similar spatial localization with the neural activity correlated with movement-related SMR modulation in the literature. Thus, we have provided a testable hypothesis that a similar mechanism may underlie SMR modulation during a motor task and during a resting state. A future study directly testing this hypothesis is warranted.

### Study Limitation

No EMG/visual monitoring was employed to rule out potential bodily movements during EEG-fMRI.

## Author Contributions

The work presented here was carried out as a collaboration among all authors. ST and JU conceived and designed the research; ST and SS performed experiments; ST analyzed data; ST, TH, and JU interpreted results of experiments; ST prepared figures and drafted the manuscript; all authors edited and revised the manuscript; all authors have read and approved the final version of the manuscript.

## Conflict of Interest Statement

The authors declare that the research was conducted in the absence of any commercial or financial relationships that could be construed as a potential conflict of interest.

## Footnotes

1 http://www.fil.ion.ucl.ac.uk/spm
